# Toxicity and Potential Pharmacological Activities in the Persian Gulf Venomous Sea Anemone, *Stichodactyla haddoni*

**Published:** 2018

**Authors:** Ziba Moghadasi, Shahla Jamili, Delavar Shahbazadeh, Kamran Pooshang Bagheri

**Affiliations:** a *Department of Marine Biology, Faculty of Marine Sciences and Technologies, Science and Research Branch, Islamic Azad University, Tehran, Iran. *; b *Iranian Fisheries Science Research Institute, Agricultural Research, Education and Extension Organization. *; c *Laboratory of Venom and Biotherapeutics Molecules, Department of Medical Biotechnology, Biotechnology Research Center, Pasteur Institute of Iran, Tehran, Iran.*

**Keywords:** *Stichodactyla haddoni*, Venom; Toxicity, Analgesic activity, The Persian Gulf sea anemone

## Abstract

Numerous proteins and peptides in venomous marine animals are potentially active molecules with pharmacological properties. Particular condition of the Persian Gulf as a closed ecosystem is a good opportunity to study of biological activities and toxicity of venomous animals. In this study, *Stichodactyla haddoni* (*S. haddoni*), a sea anemone, selected to tracing for possible pharmaceutical agents and toxicological characterization. Analgesic, edematogenic, dermonecrotic, LD50, phospholipase, and proteolytic activities of the venom were estimated. LD50 was recorded at 675 μg by intraperitoneal injection. Analgesic activitiy of crude venom on Balb/c mice at both 100 and 150 µg were dose dependent as a linear trend. Three folds increase of activity was seen at both 100 and 150 µg after 240 min comparing to activity of morphine at 200 µg. The crude venom at amount of 0.23 µg produced 50% hemolysis. The highest edematogenic activity was seen on Balb/c mice just two hours after injection for both 168 µg (157%) and 335 µg (247%). The crude venom at 675 µg made 4 mm inflammation area on rabbit skin after 3 h but the amount of 1000 µg induced 8 mm necrosis area. Potent analgesic activity of the venom was seen below its toxic dose that was very greater than the other sea anemones in the other geographical areas. The results indicate that a persistent edematogenic activity could be happened after envenomation. Instant potent edematogenic and rapid dermonecrotic activity were significant phenomena. HD50 at 0.23 µg indicates that a very potent hemolytic agent exists in the venom. The results would also be of high value to better management of envenomation. This study confirmed the great value of further studies on the Persian Gulf *S. haddoni* venom.

## Introduction

Numerous proteins, peptides, and chemical agents in the venom of venomous marine animals are potentially useful biologically active molecules with pharmacological properties. During the past decade, many studies have focused on tracing of novel drugs from marine animals (1-12). 

Among many underestimated marine derived potential drugs, some of them including a potent anti-pain drug, ziconotide, (Prialt^®^) (13), and a series of anticancer drugs like brentuximab vedotin (Adcetris^®^) (14), trabectidin (Yondelis®) (15), cytarabine (Cytosar-U^®^, Depocyt^®^) (16, 17), and vidarabine (Vira-A®) (9, 18) are approved and currently used in the market. These agents are significant successful examples of the marine drugs.

A series of drug candidates derived from marine invertebrate and vertebrate animals are in different phases of clinical studies including plitidepsin, bryostatin, hemiasterlin, and elisidepsin against lymphoma, leukemia, colon, and breast as well as other various cancers (19-22), tetrodotoxin for chronic pain (23), DMXBA for alzheimer, and schizophrenia (24), and pseudopterosin for wound healing (25). 

In this regards, venom characterization of marine venomous animals would be led to finding a new potential therapeutic agent (1, 5, 26 and 27). 

Among the Persian Gulf venomous animals, *S. haddoni*, a venomous sea anemone, selected to biochemical characterization and tracing for possible pharmaceutical agents. *Stichodactyla haddoni* is an ocean dwelling sedentary organism belonging to the family of Stichodactylidae. The venom of Stichodactylidae family could be a potential source of bioactive pharmaceutical compounds (11, 28-30). 

According to documented reports, sea anemone envenomation induces clinical signs and symptoms like paralysis, skin swelling, nausea, tingling, necrosis, vertigo, redness, and vomiting as well as cardiotoxicity and neurotoxicity (31-35).

The venom of all sea anemones is produced by specialized cells, known as nematocyst. The most nematocysts are located on the surfaces of tentacles and in lesser density on the body surface. The capsule contains a tightly wrapped and spiralized thread which is extruded under physico-chemical stimuli. 

Reference to a survey among many reports, there is no report for an approved drug extracted or derived from the venom of Stichodactylidae family yet (1, 5 and 6). 

Venom characterization of clinically important venomous animals concerning geographical distribution is a national task that could be included in the national or regional strategies for antiserum production. Furthermore, the data concluded from simulation of toxicity in animal model may have positive effect on management of envenomation by toxicity national centers and hospitals. Another possible conclusion of venom characterization is induction of an amazing insight to discovery of potential pharmaceutical agents.

Accordingly, the present study was aimed to characterization of the venom of the Persian Gulf *S. haddoni* from the point of analgesic, phospholipase, proteolytic, hemolytic, coagulation, edematogenic, lethal, and dermonecrotic activities. This study would be assisted in finding potential agents from venom components of the sea anemones and also improving clinical management of *S. haddoni* envenomation. 

## Experimental

*Reagents and media*

Casein, coomassie brilliant blue R250, lecithin (phosphatidylcholine), and triton purchased from Sigma–Aldrich Co. Thromboplastin-D and activated partial thromboplastin time (APTT) reagents were obtained from Fisher Scientific Co., USA. 

*Sample collection*

**Figure 1:**
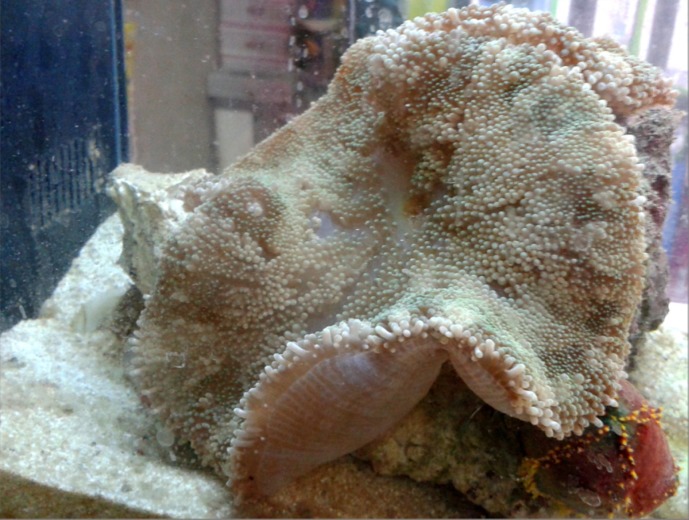
*Stichodactyla haddoni *collected from the Persian Gulf, Larak Island

**Figure 2:**
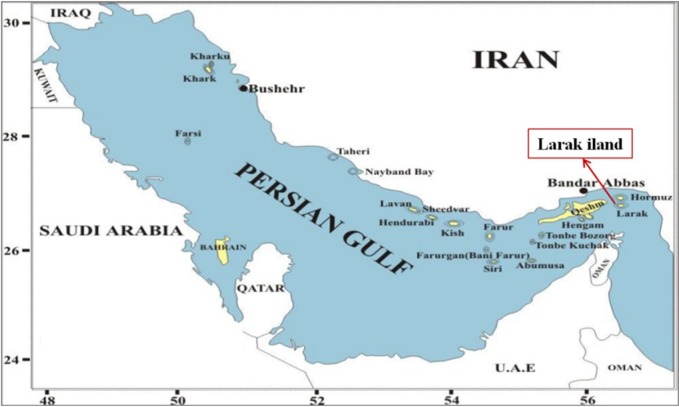
Specimen collection area. Larak Island, the Persian Gulf (26°51'12" N 56°21'20" E).

One sample of *S. haddoni* (Figure 1) weighted 9.65 g were collected manually a depth of 20 m from coastal waters of Lark island, the Persian Gulf (the south of Iran) (Figure 2) in July 2015. The specimens were kept in -20 °C and transferred to venom laboratory at Pasteur Institute of Iran.

*Venom extraction and preparation*

**Figure 3:**
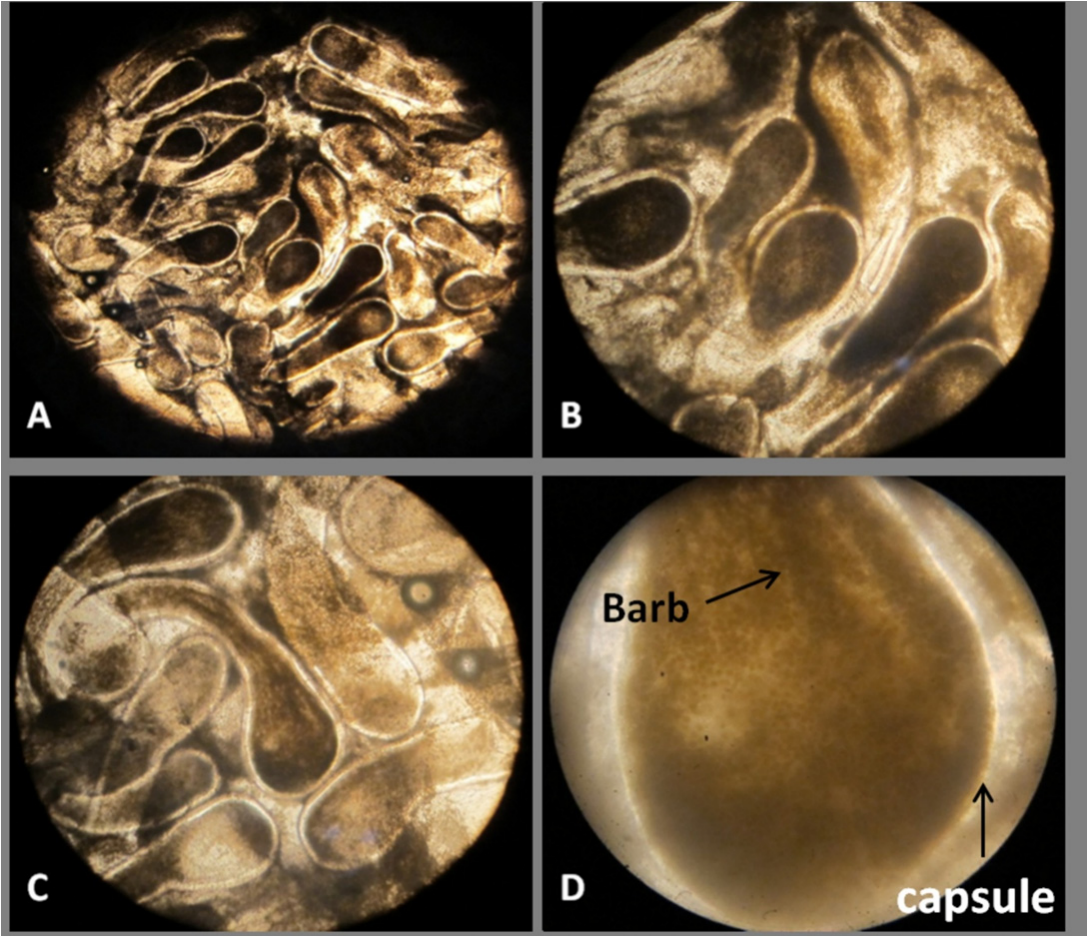
Microscopic image of the tentacles of *Stichodactyla haddoni*. Tentacles containing cnidocytes (A: 4X, B and C: 10X). Capsule and barb (D: 40X).

The frozen specimen thawed in room temperature and surface mucus layer cleaned. Tentacles (Figure 3) were removed from base by a sterile scalpel, trimmed to small pieces, and divided into two equal parts weighted 4.82 g for venom extraction. 

*Methanol extraction*

Twenty-five mL methanol solution (5%) was added to the fragmented tentacles, mixed in a vortex and incubated at 4 °C for 24 h. The solution centrifuged at 10625 g for 10 min (Sigma 1-14, Germany). Supernatant lyophilized in a freeze dryer system (Alpha 1-2 LD plus, Martin Christ Gefriertrocknungsanlagen Co., Germany). The powder resuspended in sterile water for injection and stock solution preserved at -70 °C. 

*Water extraction*

Sterile deionized water (50 mL) added to the fragmented tentacles and extraction performed as detailed above.

*Protein Estimation*

Concentration of crude venom was determined by bicinchoninic acid (BCA) protein assay method according to manufacturer instructions (iNtRON Biotechnology Co. South Korea).

*SDS-PAGE*

To determine the protein profile of extracted venom, Sodium Dodecyl, Polyacrylamide Gel Electrophoresis (SDS-PAGE) was performed on 12% polyacrylamide gel based on standard method (36).

*Lethal activity*

Lethal activity of crude venom was examined in Balb/c mice based on Spearman-Karber’s method (37). Specimen conditions for this study were approved by Ethical Committee of the Pasteur Institute of Iran (IR.PII.REC.1394.38) in accordance with EU Directive Guideline (2010/63/EU) for animal experiments. 

Briefly, different amounts of venom ranged from 50 to 675 µg were prepared in sterile deionized distilled water (DDW). Toxicity in male Balb/c mice (average weight 20–22 g) was tested by intraperitoneal injection (IP) of 100 µL of crude venom. 

Lethality was monitored during 36 h and LD50 calculated based on the following formula: 

$\log\mathrm{LD}50=\mathrm{logX}100-[\left(\frac{\mathrm{logfd}}{n}\right)\times \left(\frac{\sum T}{n}\right)]$

*Analgesic activity*

Before the anti-pain assay, each of the Balb/c mice were placed on a hot plate at 55 °C and the numbers of licking were documented during 40 sec by two observers. 

The results of this pre-experiment were considered as basic pain signal that was specific for each mouse and used for calculation of analgesic ratio. After 15 min, two doses of crude venom, 100 and 150 µg, were injected intraperitoneally into the abdomen cavity of test groups and anti-pain assay repeated every 30 min for 240 min. This experiment was performed in triplicates. Morphine (200 µg) injected as positive control and its anti-pain activity examined as the same method as test group. At the end of experiment, the percent of analgesic activity in each point of time was calculated according to our novel following math formula: 

$\mathrm{Analgesic activity}=\left[\left(\frac{\mathrm{licking number in test group in each time}}{\mathrm{licking number before injection in test group}}\right)\times 100\right]-\mathrm{100}$

*Proteolytic activity*

Proteolytic activity was based on a new rapid method innovated by Dr. Kamran Pooshang Bagheri (38). Briefly, test solutions (100 µL) containing ascending amounts of venom including 50, 25, 12.5, 6.25, 3.125, 1.56, 0.78, and 0.39 µg were incubated with casein solution (0.5%, containing 0.008 M calcium chloride at pH 7.5) for 2 h at 37 °C. To stopping the reaction and instant staining, coomassie brilliant blue R 250 (50 µL, 1X) was added to the solution. Optical density was read at 595 nm in a microplate spectrophotometer (EPOCH, BioTeK Co., USA) and protein concentration calculated reference to BSA standard curve. Casein (0.5%, containing 0.008 M calcium chloride at pH 7.5) used as control. 

*Phospholipase activity*

Phospholipase activity of the crude venom was determined by colorimetric method (39) with some modifications. Briefly, serial amounts of venom from 0.78 to 100 µg added to a microplate and then, substrate solution (100 µL) was added to each well and incubated at 37 °C for 30 min. Optical density was read at 550 nm in a microplate spectrophotometer (EPOCH, BioTek Co., USA). *Macrovipera lebetina* (viper snake) and *Apis melifera* meda (honeybee) venom were used as positive controls. Deionized water was used as negative control. 

*Edematogenic activity*

The ratio of 1/2 and 1/4 of LD50 dose selected to examine edematogenic activity in Balb/c mice (male, 20 g). Based on these ratios, two quantities of *S. haddoni* venom, 168 and 336 µg, prepared in sterile 0.9% NaCl (W/V), and 30 µL was injected into the subplantar region of the left hind paw. The right hind paw received the equal volume of sterile saline alone and served as the control.

The diameters of left paws were measured using caliper at 0, 0.5, 1, 2, 3, 4, 24, 48, 72 h, 10 days, and 30 days after venom administration. The percentage of edema was calculated based on the following formula: 

$\left[\frac{\mathrm{Thickness of the left paw}-\mathrm{Thickness of the right paw}}{\mathrm{Thicknessof the right paw}}\right]\times 100$

*Hemolytic activity*

This assay was performed as described before (38). Fresh human blood was drawn by venous puncture using heparinized tubes. Complete blood count (CBC) assay was performed to verify the health status of the donor. Erythrocytes were washed three times with phosphate-buffered saline (pH 7.4) and a suspension of 2% RBC was prepared in normal saline. Serial amounts of venom prepared in a microplate ranged from 100 to 0.78 µg in 100 µL normal saline and 2% RBC suspension (100 µL) added to each well and incubated for 1 h at 37 °C. The samples were than centrifuged for 10 min at 1664 g (Sigma 3-18k), and the absorbance of the supernatant was determined at 540 nm. 

Normal saline and triton X100 were used as negative and positive control respectively. The percent of hemolysis was calculated as follows: 

$\mathrm{Hemolysis}\left(\%\right)=\left[\frac{\mathrm{OD}\mathrm{test}-\mathrm{OD}\mathrm{negative}\mathrm{control}}{\mathrm{OD}\mathrm{positive}\mathrm{control}-\mathrm{OD}\mathrm{negative}\mathrm{control}}\right]\times 100$

*Dermonecrotic activity*

Dermonecrosis activity of the venom was checked on New Zealand rabbits. Different amounts of *S. haddoni* venom including 200, 675, and 1000 µg prepared in sterile NaCl solution (0.9% W/V). Two-hundred µL of each amount was injected into the shaved areas of back skin intradermally. The equal volume of sterile saline served as negative control. Inflamed and necrotic area was measured using a ruler after 3, 24, and 48 h.

*Determination of coagulation activity on human plasma*

Effect of venom on extrinsic and intrinsic pathways of coagulation were evaluated in human plasma by prothrombin time (PT) and partial thromboplastin time (PTT) respectively.

*Prothrombin time assay*

Plasma was collected from a healthy donor. To confirm the health status of the donor, PT and PTT assay were performed and controlled with standard control reagent recommended by manufacturer (Fisher Scientific Co., USA). 

Different amounts of *S. haddoni* venom (7, 14, and 28 µg) was added to citrated plasma (100 μL) and incubated for 5 min at 37 °C in a water bath. Thromboplastin-D (200 μL) was then added and clotting time was recorded by a digital timer. Citrated plasma and PT control reagent were used as normal control. 

*Partial thromboplastin time assay*

Different amounts of *S. haddoni* venom (25, 50, and 100 µg) were added to 100 μL of citrated plasma and incubated for 5 min at 37 °C. One-hundred μL of a PTT reagent was then added and mix together. One-hundred μL of 0.1 M calcium chloride was then added and clotting time was recorded as above. One-hundred µL of a PTT reagent was added to 100 μL of citrated plasma (incubated for 1 min at 37 °C), followed by the addition of 100 µL of calcium chloride (preheated to 37 °C) and the clotting time was measured. Citrated plasma and PTT control reagent used as normal control.

*Statistical evaluations*

To describe the correlation between the examined doses and percent of activities or time dependency, linear regression test was performed by SPSS software (Ver. 22). 

## Results

*SDS-PAGE*

**Figure 4:**
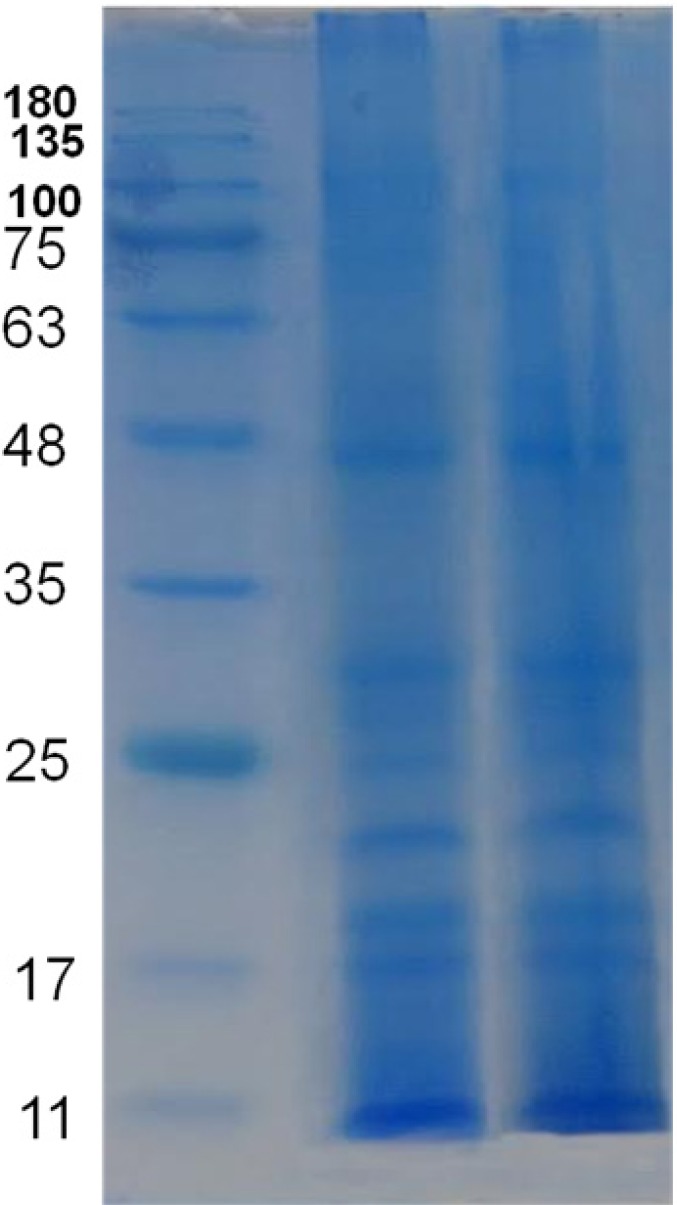
Electrophoretic profile of the Persian Gulf *S. haddoni*. From left to right, Lane1: Molecular weight marker, Lane2: Methanolic extract of *S. haddoni* crude venom. Lane 3: Water extract of *S. haddoni* crude venom. According to the results, no difference was seen between protein profile of water extract and methanol extract therefore a mixture of both extracts was used for characterization of the venom

SDS-PAGE results showed 12 separate bands and the molecular weight of observed protein ranged approximately from 8 to 250 kDa (Figure 4). According to the results, no difference was seen between protein profile of water extract and methanolic extract therefore a mixture of both extracts was used for characterization of the venom. 

*LD50*

Intraperitoneal administration of 675 µg of crude venom was induced 50% lethality in test group. At 450 µg, significant lethargy, weak movement, and tachycardia were seen. At 200 and 300 µg, no signs were seen. 

*Analgesic activity*

**Figure 5:**
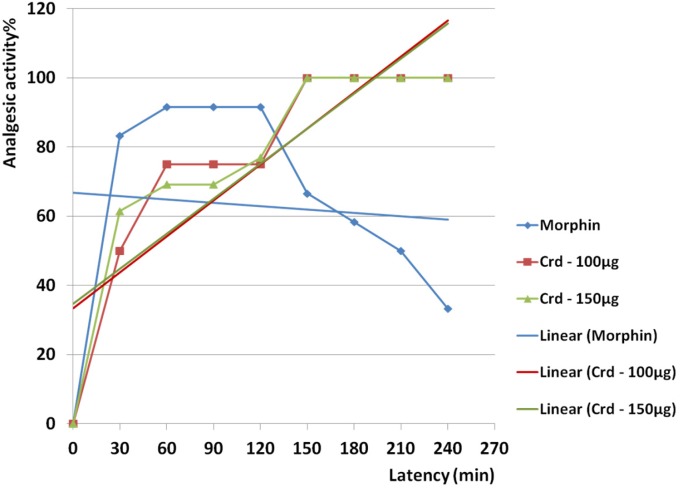
Analgesic activity of crude venom of *S. haddoni* and morphine on Balb/c mice in hot plate assay. Analgesic activity of crude venom at the amounts of 100 and 150 µg was greater than morphine (200 µg). Anti-pain activities of both amounts of crude venom were dose dependent and slope of activity were highly increased from zero point up to 60 min. Activity of 100 µg crude venom was constant during the time between 60 and 120 min. This phenomenon was seen for 150 µg crude venom too but from 60 and 90 min. After 90 min of crude venom administration, analgesic behavior of both 100 and 150 µg was similar up to 240 min. From 90 to 150 min for 150 µg and from 120 to 150 min for 100 µg, slope of activity were intensely raised and activity documented as 100% at 150 min. One-hundred percent analgesic activity was constant during 90 min up to 240 min. According to calculated R^2^, both 100 (R^2 ^= 0.744) and 150 µg (R^2 ^= 0.735) reduced pain as a linear behavior.

Analgesic activity of crude venom at the amounts of 100 and 150 µg was greater than morphine. Anti-pain activities of both amounts of crude venom were dose dependent and slope of activity were highly increased from zero point up to 60 min. Activity of 100 µg crude venom was constant during the time period between 60 and 120 min. This phenomenon was seen for 150 µg crude venom too but from 60 and 90 min. After 90 min of crude venom administration, analgesic behavior of both 100 and 150 µg was similar up to 240 min. From 90 to 150 min for 150 µg and from 120 to 150 min for 100 µg, slope of activity were intensely raised and activity documented as 100% at 150 min. One-hundred percent analgesic activity was constant during 90 min up to 240 min. According to calculated R^2^, both 100 (R^2 ^= 0.744) and 150 µg (R^2 ^= 0.735) reduced pain as a linear behavior (Figure 5).

Analgesic activity of morphine was seen faster and greater than both amounts of crude venom during the first 60 min. Activity of morphine was constant from 60 to 120 min during 60 min (91.6%) and slope of activity was rapidly dropped to 33.3% from 120 to 240 min. Based on calculated R^2^ for morphine (R^2^ = 0.007), the activity was not dose dependent (Figure 5). 

*Comparison of analgesic activity of crude venom and morphine*

**Figure 6:**
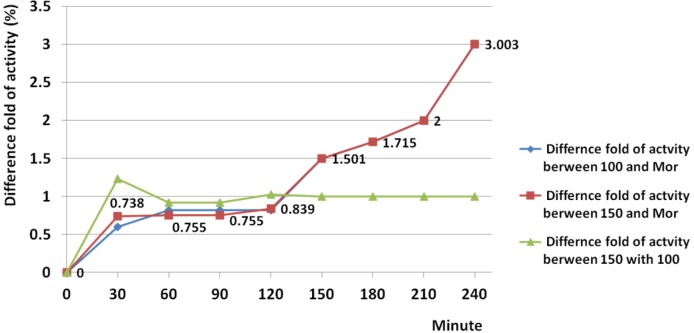
Comparison of analgesic activity of crude venom and morphine and examined doses. According to results, both 150 and 100 µg had lesser activity than morphine from start point up to 120 min. After 128 min, the activity was significantly raised up to 240 min. Threefold increase of activity was seen at 240 min comparing to activity of morphine, at both 150 and 100 µg.

**Figure 7:**
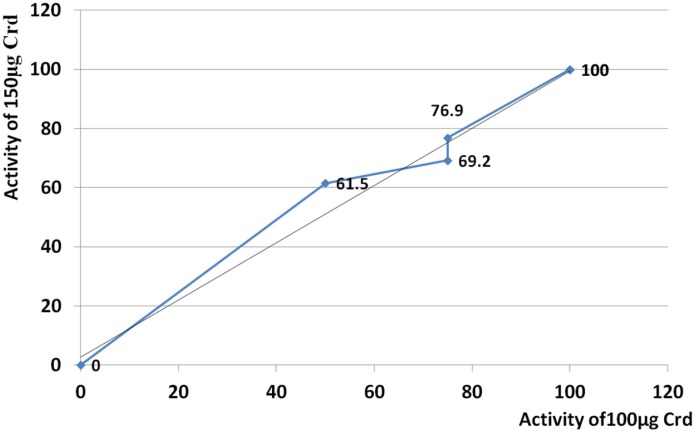
Comparison of the trend of analgesic activity between 100 and 150 µg. Based on the regression analysis, both examined doses induced similar trend of activity (R^2 ^= 0.976). Crude venom was abbreviated as “Crd”.

This comparison evaluated generally and also performed at all points of time. According to results, both 150 and 100 µg had lesser activity than morphine from start point up to 120 min. After 128 min, the activity of both doses was significantly raised up to 240 min. Threefold increase of activity was seen at 240 min comparing to activity of morphine, at both 150 and 100 µg (Figure 6). Based on the regression analysis, both examined doses induced similar trend of activity (R^2 ^= 0.976) (Figure 7).

*Hemolysis assay*

**Figure 8:**
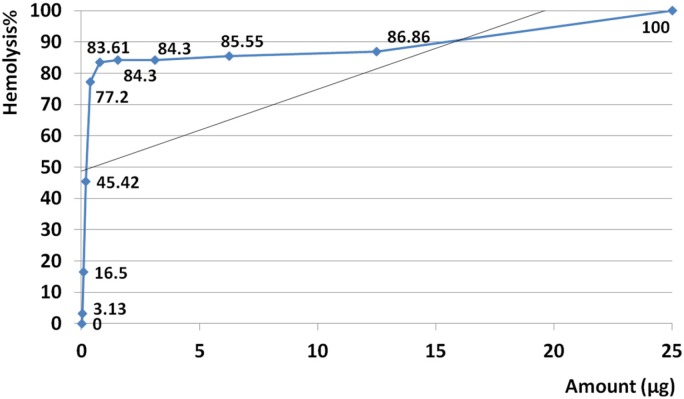
Hemolytic activity of *S. haddoni* venom. The amount of 25 µg crude venom produced 100% hemolysis and HD50 identified at 0.23 µg. Based on regression analysis, no general linearity was seen at examined amounts of venom ranged from 0.023 to 25 µg. This indicates that hemolysis activity was generally not dose dependent at estimated range (R^2 ^= 0.299).

The amount of 25 µg crude venom produced 100% hemolysis and HD50 was identified at 0.23 µg. Based on regression analysis, no linearity was seen at examined amounts of venom ranging from 0.023 to 25 µg. This means that hemolysis activity was not seen as dose dependent manner at the examined range (R^2 ^= 0.299) (Figure 8).

**Figure 9:**
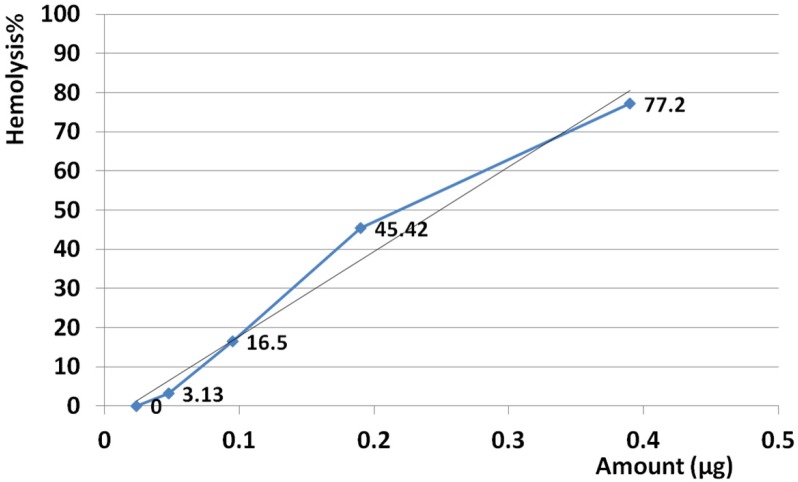
Slope of hemolysis activity intensely increased from 3.13 to 77.2% that this raising trend corresponding to amounts ranged from 0.023 µg (23 ng) up to 0.39 µg (390 ng). According to calculated R^2^ high linearity was seen from 0.023 µg up to 0.39 µg (R^2 ^= 0.978). This result confirmed that hemolytic activity strangely was linear at the range of 0.023-0.39 µg.

**Figure 10:**
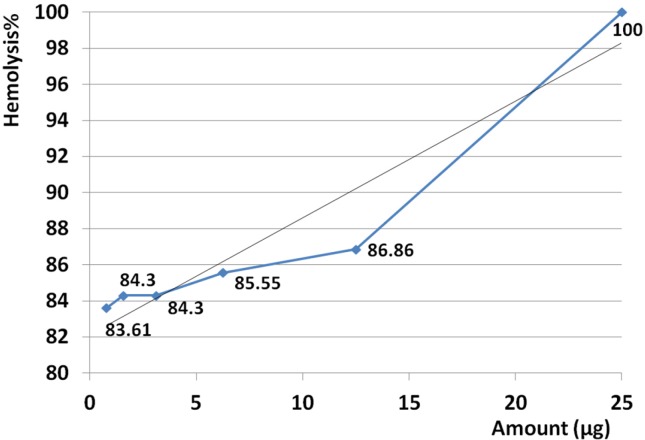
Slope of hemolysis activity gradually raised from 83.6 to 100% that was corresponded to the amounts ranged from 0.78 to 25 µg. Reference to calculated R^2^, high linearity was seen at examined range of doses (R^2 ^= 0.914). This result demonstrates that hemolytic activity strangely was linear at the range of 0.78 to 25 µg.

Slope of hemolysis activity intensely increased from 3.13 to 77.2% that this raising trend corresponding to amounts ranged from 0.023 µg up to 0.39 µg. According to calculated R^2^ high linearity was seen at this range (R^2 ^= 0.978) (Figure 9). Slope of hemolysis activity gradually raised from 83.6 to 100% that was corresponding to the amounts ranged from 0.78 to 25 µg. Reference to calculated R^2 ^, high linearity was seen at this range (R^2 ^= 0.914) (Figure 10).

*Phospholipase activity*

The venom did not show phospholipase activity up to 100 µg of the crude venom. 

*Proteolytic activity*

The venom did not induce proteolytic activity up to 50 µg of crude venom.

*Edematogenic activity*

Edematogenic activity was seen for both examined doses of the crude venom. The crude venom at 335 µg induced greater edematogenic activity than 168 µg. The highest activity was seen just two hours after injection for both 168 µg (157%) and 335 µg (247%). 

**Figure 11:**
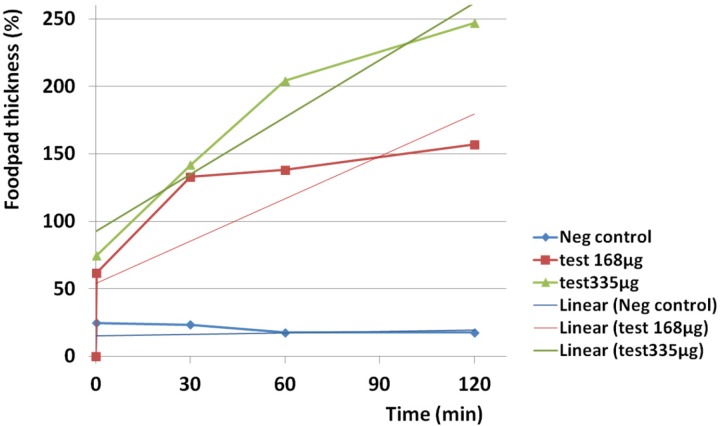
Estimation of edematogenic activity of *S. haddoni* crude venom on Balb/c mice. The highest activity was seen just two hours after injection for both 168 µg (157%) and 335 µg (247%). The crude venom at 335 µg induced greater edematogenic activity than 168 µg. The slope of activity was more rapidly raised at 335 µg and showed a linear trend (R^2 ^= 0.921) in comparison to 168 µg (R^2 ^= 0.64) after two hours.

The slope of activity was rapidly increased at 335 µg and showed a linear trend (R^2 ^= 0.921) in comparison to 168 µg (R^2 ^= 0.64) after two hours (Figure 11).

**Figure 12:**
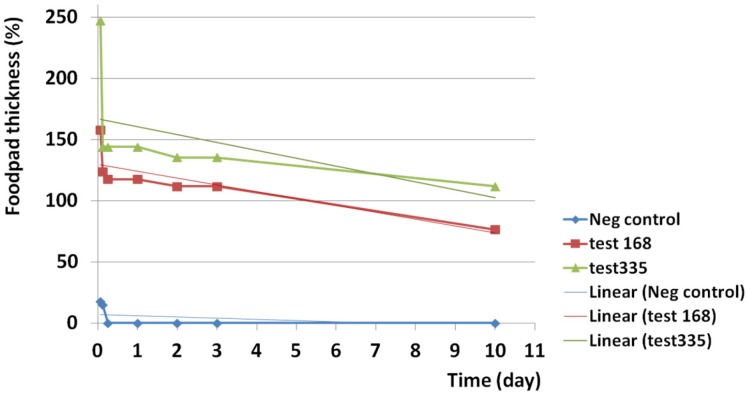
Edematogenic activity of *S. haddoni* crude venom during a 10 days period. Edematogenic activities of 168 and 335 µg of crude venom was slowly reduced to 76.4 and 111.7% after 10 days respectively. Reduction trend for 168 µg was linear and time dependent (R^2 ^= 0.693) but at 335 µg edema had not time dependent behavior. The results indicate that a persistent edematogenic activity could be happened after envenomation by the Persian Gulf *S. haddoni*.

Edematogenic activities of 168 and 335 µg of crude venom was slowly reduced to 76.4 and 111.7% after 10 days respectively. Reduction trend for 168 µg was linear and time dependent (R^2 ^= 0.693) but at 335 µg edema had not time dependent behavior (R^2 ^= 0.273). The results indicate that a persistent edematogenic activity could be happened after envenomation by the Persian Gulf *S. haddoni* (Figure 12). 

**Figure 13:**
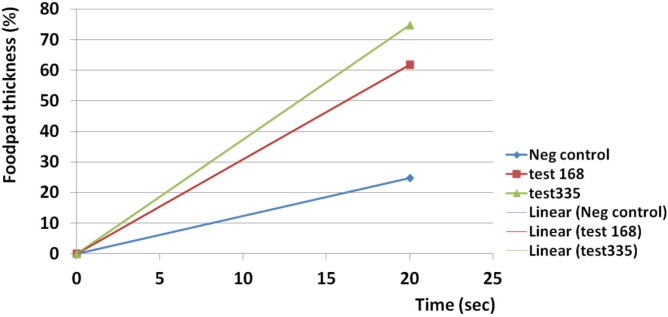
Instant edematogenic activity of *S. haddoni* crude venom after 20 sec. Injection of both amounts of crude venom at 168 and 335 µg induced immediate 61.7 and 74.7% swelling on subplantar region of Balb/c mice respectively as a high linear trend (R^2 ^= 1). Induction of edema at 335 µg was greater than 168 µg. A linear trend was seen in control group (R^2 ^= 1) but the amount of swelling was lesser than test groups.

Injection of both amounts of crude venom at 168 and 335 µg induced immediate 61.7 and 74.7% swelling on subplantar region of Balb/c mice respectively as a high linear trend (R^2 ^= 1). Induction of edema for 335 µg was greater than 168 µg. A linear trend was seen in control group (R^2 ^= 1) but the amount of swelling was lesser than test groups (Figure 13). 

*Coagulation assay*

No coagulation activity was seen in PT and PTT assays. 

*Dermonecrotic activity*

**Figure 14:**
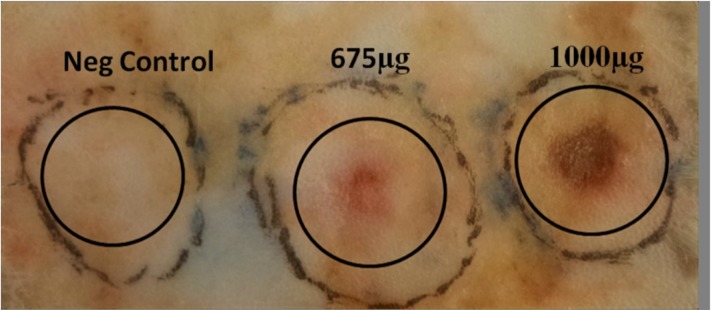
Dermonecrotic activity of *S. haddoni* crude venom on rabbit skin. Crude venom at 675 µg made 4 mm inflammation on rabbit skin after 3 h but 1000 µg induced 8 mm necrosis area.

A very fast acting dermonecrotic and inflammation activity was seen during 3 h after injection of 675 and 1000 µg of crude venom. Crude venom at 675 µg made 4 mm inflammation area on rabbit skin after 3 h but 1000 µg induced 8 mm necrosis area (Figure 14). 

**Figure 15:**
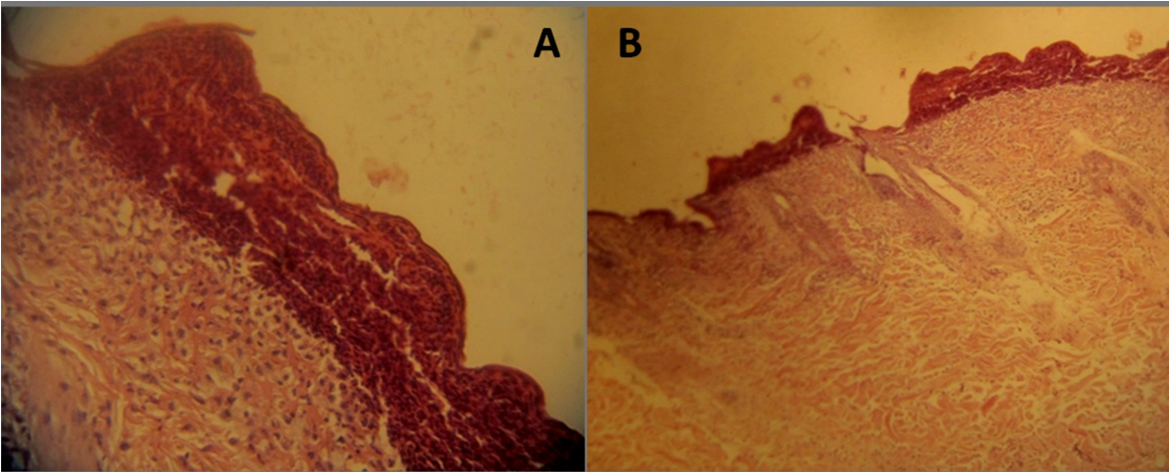
Pathological events induced by intradermal injection of *S. haddoni* crude venom on rabbit skin. Necrotic area is shown in surface of skin. (A and B) Infiltration of lymphocytes and necrosis were seen by injection of 1000 µg and 675 µg of venom. (A: 40X, B: 10X).

Infiltration of lymphocytes and necrosis were seen after injection of 1000 µg (A) and 675 µg (B) of venom (Figures 15A and 15B).

## Discussion

During the past decades, many geographical regions have been traced for potential marine pharmaceutical agents while a few studies have been documented about characterization of the Persian Gulf venomous animals. The Persian Gulf is a closed important ecosystem in which many genera have evolved apart from the other similar animals. This particular condition is a good opportunity to study of biological activities in venomous animals concerning the significant value of tracing for potential pharmaceutical agents. From the other point of view, study of the venom toxicity would be assisted in the development of more effective treatment protocols in possible envenomations. During the past decade, many studies have focused on tracing of novel drugs from marine animals. Among many marine derived potential drugs, Ziconotide, a potent anti-pain drug, is a good example that was found in the venom of the fish-eating marine snail, *Conus magus*.

*S. haddoni* is one of the venomous animals of the Persian Gulf and characterization of its biological activity and toxicity are of great value concerning discovery of new potential pharmaceutical agents as well as study of toxicity of the crude venom.

According to LD50 results, IP injection of the Persian gulf *S. haddoni* venom is not toxic up to 150 µg and moderately toxic up to 450 µg. LD50 was seen at 33.75 mg/kg (675 µg/mouse). LD50 of the other sea anemones (IP injection) including *Stichodactyla mertensii* (*S. mertensii*) (40), *Gyrostoma helianthus *(*G.* *helianthus*) (41), and *Bartholomea annulata* (*B. annulata*) (42) were 108.24, 29, and 700.7 mg/kg respectively. Comparison of LD50 of the other genus of Stichodactylidae family with the Persian gulf* S. haddoni* was variably different. 

Analgesic activity of morphine was seen faster and greater than both amounts of crude venom during the first 60 min. Activity of morphine was constant from 60 to 120 min during 60 min (91.6%) and slope of activity was rapidly dropped to 33.3% from 120 to 240 min. Based of calculated R^2^ for morphine (R^2 ^= 0.007), the activity was not dose dependent. Crude venom is abbreviated as “Crd”.

.

Hemolysis activity on human erythrocytes was not seen in dose dependent manner at estimated doses (R^2 ^= 0.299) but at two distinct ranges of 0.023-0.39 µg (R^2 ^= 0.978) and 0.78-25 µg (R^2 ^= 0.914) the activity was dose dependent and linearity was significant. Similarly, Subraminan *et al.* (2011) showed the hemolytic activity of *Paracondactylis indicus* (*P. indicus*) and *Paracondactylis sinensis* (*P. sinensis*) respectively against chicken erythrocytes and also *Heteractis magnifica* (*H. magnifica*) and *S. hadonii* against chicken and goat erythrocytes (33). Sudharsan *et al.* (2013) reported hemolytic activity of *S. mertensii* methanolic crude venom extract on chicken and human erythrocytes too (40). 

One-hundred percent of analgesic activity was seen at both doses after 150 min. This activity was constant up to 240 min while activity of morphine did not reach to 100% and it dropped rapidly after 120 min. Three fold increase of activity was seen at 240 min comparing to activity of morphine, at both 150 and 100 µg. Similar significant linear trend of analgesic activity was seen at both examined doses based on the regression analyses. We successfully measured the percent of analgesic activity by our novel formula and the trend of activity discussed based on this estimation. 

As none of documented studies reported based on percent of activity, we could not able to compare our results with the others. 

Sudharsan *et al.* (2013) reported the analgesic activity of *S. mertensii* tentacle methanolic extract (312.5 µg) in tail flick assay (40). Suberamanian *et al.* (2011) reported analgesic activity of the crude nematocyst extract of different sea anemones including *P. indicus*, *P. sinensis*, *H. magnifica,* and *S. haddoni* showing analgesic activity at 2 mg/mL (40 µg) by hot plate assay (33) up to 120 sec but the assay time in our study was conducted up to 240 min. The different analgesic activity of the Persian Gulf *S. haddoni* is an amazing report and demonstrates that different geographical distributions has different effects on biological activity of similar genus and species as Suberamanian *et al*. collected *S. haddoni *from Mandapam coast, India. 

Suganthi *et al.* (2011) exhibited maximum analgesic activity of the aqueous extract of jelly fish *Crambionella stuhalmanni* (*C. stuhalmanni*) and *Chrysaora Quinquecirrha *(*C. Quinquecirrha*) (Cnidaria phyllum) at 5 mg/mL (100 µg) by hot plate assay after two hours (43). In comparison, our maximum activity (100%) was obtained after 150 min. This indicates that activity of *S. haddoni* is slightly slower than *C. stuhalmanni* and *C. Quinquecirrha*. 

Immediate edema after intradermal injection of venom indicates the existence of a very potent edematogenic molecule in the venom. Maximum thickness was seen after two hours for both 168 and 335 µg of crude venom and edema decreased very slowly during 10 days of follow up measurements to 76.4 and 111.7% respectively. The results indicate that a persistent edematogenic activity could be happened after envenomation by the Persian Gulf *S. haddoni*. Subramanian *et al*. in 2011 also showed edematogenic activity of the crude extract of *P. sinensis* and *S. haddoni* collected from east coast of India (33). 

No proteolysis activity was seen against casein for *S. haddoni.* González *et al.* (2016) showed that in accordance to our report, many sea anemones including *Bunodosoma granulifera*, *Stichodactyla helianthus* and other cnidaria such as *Plexaura homomalla, Physalia physalis, *and* Zoanthus sociatus* had no proteolytic activity. Adhikari *et al.* (2007) and González *et al.* (2016) reported proteolytic activity of the crude venom of *P. indicus Dave* and *Condylactis gigantica* (*C. gigantean*) (44, 45). 

No Phospholipase activity was seen in this study but some phospholipase A2 (PLA2s) was isolated from the venoms of other Sea anemones like *Bunodosoma caissarum*, *C. gigantean*, *Urticina crassicornis,* and *Adamsia carciniopados* (46-49).

No coagulation activity was seen for *S. haddoni*. Our report is in accordance with Annadurai *et al.* study in 2012 in which methanolic and aqueous extracts of sea anemones *i.e*. *Heteractis magnifica* and *S. haddoni* had no coagulation activity in both APTT and PT assays (50).

A very fast acting dermonecrotic activity was seen during 3 h after intradermal injection of 675 and 1000 µg of crude venom. This is the first report of a rapid dermonecrolysis by *S. haddoni* venom. We suggest examining its necrotic activity against skin tumors like melanoma. 

Although purification and characterization of venom molecules solely are very useful, since a mixture of many molecules in the venom induce their activity and toxicity beside together, evaluation of crude venom should also be performed and seems that this approach is more real and applicable to determination of toxic activities. This re-evaluation approach would be suggested to characterization of the other marine venomous animals. 

## Conclusion

Potent analgesic activity of the venom below its toxic dose pointed to existence of a valuable anti-pain molecule in the crude venom. Similar significant linear trend of analgesic activity was seen at both examined doses based on the regression analyses. The results indicate that a persistent edematogenic activity could be happened after envenomation by the Persian Gulf *S. haddoni*. Instant edema and rapid dermonecrotic activity were the significant phenomena after intradermal injection of the venom. HD50 at 0.23 µg indicates the existence of a very potent hemolytic agent in the venom.

This report, like the history of anti-pain activity in conus venom that led to discovery of ziconotide as a potent drug, can induce an interesting insight in other scientists to discovery of valuable pharmaceutical agents from similar marine animals.

The data resulting from simulation of *S. haddoni* envenomation would also be of high value to better management of clinical conditions and following up treatment strategies as well. 

According to our results, a high potent peripheral analgesic agent is in the crude venom of the Persian Gulf *S. haddoni* that its activity was very greater than the other sea anemones in the other geographical areas. It is the first report on instant potent edematogenic, potent hemolytic, and rapid dermonecrotic activities of the Persian Gulf sea anemone venom, *Stichodactyla haddoni*. This study confirmed the great value of further studies on the Persian Gulf *S. haddoni* venom. 
